# Zn^2+^ Interaction with Amyloid-B: Affinity and Speciation

**DOI:** 10.3390/molecules24152796

**Published:** 2019-07-31

**Authors:** Giuseppe Arena, Enrico Rizzarelli

**Affiliations:** 1 Department of Chemical Sciences, University of Catania, Viale Andrea Doria 6, 95125 Catania, Italy; 2 Consorzio Interuniversitario di Ricerca in Chimica dei Metalli nei Sistemi Biologici (C.I.R.C.M.S.B.), 70125 Bari, Italy; 3 Institute of Crystallography—CNR—UOS Catania, Via Paolo Gaifami 9, 95126 Catania, Italy

**Keywords:** speciation, amyloid-β, Zn^2+^, affinity

## Abstract

Conflicting values, obtained by different techniques and often under different experimental conditions have been reported on the affinity of Zn^2+^ for amyloid-β, that is recognized as the major interaction responsible for Alzheimer’s disease. Here, we compare the approaches employed so far, i.e., the evaluation of K_d_ and the determination of the stability constants to quantitatively express the affinity of Zn^2+^ for the amyloid-β peptide, evidencing the pros and cons of the two approaches. We also comment on the different techniques and conditions employed that may lead to divergent data. Through the analysis of the species distribution obtained for two selected examples, we show the implications that the speciation, based on stoichiometric constants rather than on K_d_, may have on data interpretation. The paper also demonstrates that the problem is further complicated by the occurrence of multiple equilibria over a relatively narrow pH range.

## 1. Introduction

As of 2018, there were over 50 million people worldwide with dementia, more than 50% of whom lived in low and middle-income countries. This figure is forecast to double by 2030 (82 million people) and more than triple by 2050 (152 million people). It is estimated that, around the world, there will be a new case every three seconds [1]. Much of this increase will be in rapidly developing and heavily populated regions such as China, India and Latin America. Already 58% of people with dementia live in low and middle-income countries, but by 2050 this will rise to 68% [2]. Dementia primarily affects older people. This is particularly relevant to countries like China, India, and their south Asian and western Pacific neighbors, that have the fastest growing elderly population but also this poses major problems worldwide since the world’s population is ageing. Up to the age of 65, dementia develops in only about one person in 1000. The chance of having the condition rises sharply with age to one person in 20 over the age of 65. Over the age of 80, this figure increases to one person in five [3]. As to the economic burden, the total estimated worldwide cost of dementia in 2018 was 1 trillion US $, and this figure is expected to rise to 2 trillion US $ by 2030, which represents more than 1% of global GDP [2].

Noteworthily, according to a 2018 report about two thirds of the 50 million people suffering from dementia have Alzheimer’s disease (AD), a progressive and devastating neurodegenerative brain disorder first described in 1906, that is also the most common cause of dementia in elderly people [1]. AD is characterized by the brain deposition of neurofibrillary tangles and senile plaques, a hallmark of this pathological disorder. Plaques consist mainly of insoluble amyloid-β (Aβ) fibril deposits [4,5,6]. The amyloid peptides (Aβ) are generated by the proteolytic action of α-, β- and γ- secretases on the large transmembrane amyloid precursor protein (APP) [7,8,9,10,11,12] and contain predominantly forty (Aβ(1–40)) and forty two (Aβ(1–42)) aminoacid residues; though less abundant, Aβ(1–42) is more neurotoxic than Aβ(1–40) [13,14,15,16].

The observation that high concentrations of metal ions are co-localized in the core of Alzheimer’s amyloid plaques has generated great interest in the effects of metal ions on β peptide misfolding and aggregation. Exposure to a number of metal ions is considered a risk factor for the onset of the disease; some metal ions accelerate protein aggregation, stabilize amyloid fibrils, and increase the neurotoxic effects of Aβ peptides in vitro [17]. Metal ions belonging to the *d* block like zinc, copper, and iron have been thought to be pathogenic agents in AD owing to the accumulation of these metals in amyloid deposits [18,19,20] and in the cortical tissues of AD patients [21]. In fact, it has been shown that these metals induce Aβ aggregation [22,23] and fibril formation [24,25].

Thus, it is not surprising that the interaction of transition metal ions with the Aβ peptides has attracted a considerable attention in recent years due to its impact on AD; in particular, a large number of reports, some of which contradicting one another, indicate that Cu and Zn have significant effects on the Aβ peptide aggregation and the stabilization of neurotoxic soluble Aβ oligomers [10]. There is still some debate on whether Aβ aggregation is the cause or only a consequence of AD and whether the oligomers are the toxic species responsible for synaptic dysfunction and neuronal cell loss in AD [26,27]. Lee et al. found that zinc ions are able to more effectively destabilize fibril structures than copper ions; according to this study, Zn^2+^ ions would promote the formation and stability of Aβ oligomers, whereas they reduce the stability of Aβ fibrils [28]. Evidence shows that the presence of Zn^2+^ can avoid [29] or delay the conversion of Aβ(1–40) into fibrils [30] but also rapidly promotes Aβ(1–40) aggregation to oligomeric species [22,31]; Mannini et al. suggest that the latter process results from the redirection of Aβ(1–40) aggregation as a result of intermediate species becoming kinetically trapped and no longer being capable of forming fibrils [32].

Previously we reviewed the affinity of Cu^2+^ to Aβ and examined the implications that a correct speciation may have on the interpretation of data obtained through different techniques [33]. In the present paper, we focus on the coordination of Zn^2+^ to Aβ and discuss the possible implications of species distribution on metal concentration-dependent effects on Aβ aggregates and their toxicities.

## 2. Zn^2+^ Interaction with Amyloid-β

The complexity of biological systems makes it difficult to quantitate interactions between metal ions and biomolecules directly in vivo. A viable route is to determine the affinity of a metal ion to a biomolecule in vitro and to extend the information obtained in vitro to in vivo conditions; the in vitro study should be carried out in conditions approaching the in vivo conditions as much as the specific technique permits. In our specific case, such a route is severely hampered by the poor solubility of Aβ in water. Consequently, fragments of the whole protein have to be employed which retain/model the binding characteristics of the native molecule; this becomes an even greater challenge when dealing with metal complexes, that often are much less soluble than the biomolecule itself.

### 2.1. Aβ(1–16)-Peg

**Figure 1:**
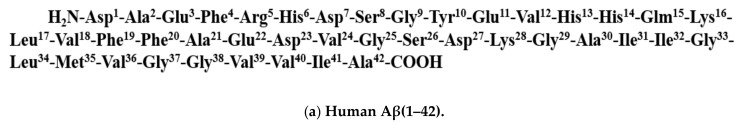

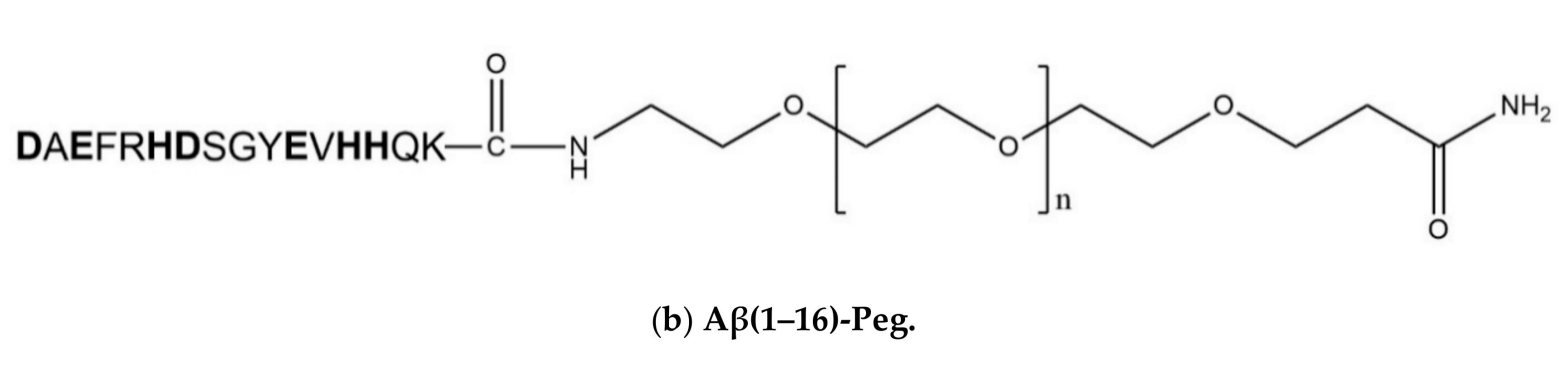
Aminoacid sequence of Aβ(1–42) (**a**) and schematic representation of Aβ(1–16)-PEG (**b**)

Amyloid-β consists of a mixture of peptides containing 39–42 aminoacid residues (see Introduction). The aminoacid sequence of human Aβ(1–42) is reported in Figure 1a.

Neither one of the amyloid-β main peptides (Aβ(1–40) and Aβ(1–42)) are soluble enough to allow for an investigation by potentiometry, the technique of choice for a reliable speciation, to see how the species concentration changes with pH or, in more general terms, with the concentration of the titrant. Thus shorter peptides that reproduce and, at least partly, retain the binding characteristics of these longer fragments have to be used. Although there are some discrepancies on the speciation/coordination mode of the most investigated metal ions (i.e., Cu^2+^ and Zn^2+^) [26,33,34], it is now well-established that in amyloid-β, the metal binding sites are located in the N-terminal hydrophilic region encompassing the amino acid residues 1–16 (Aβ(1–16)) [35,36]. Unfortunately, neither the Cu^2+^ nor Zn^2+^ complexes with the peptide reportedly containing the metal binding sites (i.e., Aβ(1–16)) can be fully characterized in aqueous solution due the formation of precipitates that prevent scanning a wide range of metal:ligand ratios. In order to overcome such a major obstacle, Aβ(1-16) has been derivatized by attaching a polyethylene glycol (PEG) chain to the C-terminus thus rendering the peptide soluble in water. It has been shown that Aβ(1–16)-PEG (Figure 1b) forms with both Cu^2+^ and Zn^2+^ complexes soluble enough to allow for a detailed potentiometric and spectroscopic characterization of the Aβ(1–16)PEG-metal ion systems [34,37]. Donor atoms potentially involved in the coordination to the metal ion are indicated in bold in Figure 1b.

### 2.2. Zn^2+^ in Alzheimer’s Disease

Zinc is an essential nutrient and the second most abundant trace element in the body [38,39]. It has a wide range of biologically relevant functions including the regulation of gene expression, protein synthesis, and cellular signaling [40]. The alteration of zinc homeostasis is involved in neurological diseases such as Alzheimer’s disease, Parkinson’s disease, and amyotrophic lateral sclerosis. According to some findings, zinc would reduce oxidative stress by binding to thiol groups, decreasing their oxidation [41,42]. Zinc dysregulation is reportedly involved in two types of neuropathology: (i) Alzheimer’s disease, and (ii) the so-called ‘excitotoxicity’ which injures neurons after ischemia, hemorrhage, seizures, or mechanical brain traumas and also affects the rate and severity of AD pathophysiology [43]. Zinc may reach concentration values as high as 1 mM in AD plaques of patients [18]. The role of zinc in amyloid fibrils formation has also been demonstrated by experiments showing that the solubilization of Aβ from post-mortem brain tissue was significantly increased by suitable Zn^2+^ chelators [44]. Although Zn^2+^ binding to Aβ is well established, the effects of such a binding, in terms of metal-dependent aggregation and toxicity, are still controversial. High concentrations promote Aβ-induced toxicity both in vitro [45] and in vivo [46]. According to others, low levels of this metal ion reduce Aβ toxicity and thus exert a neuroprotective effect [47,48,49,50] Thus, zinc would have concentration-dependent effects that may also be linked to the number of Zn^2+^ ions bound to Aβ [34].

## 3. Zn^2+^ Affinity for Aβ

The key parameter in the interaction of Zn^2+^ with Aβ is the affinity for the ligand(s) of interest. The determination of the stability constants of the complex(es) resulting from the binding of a metal ion (Zn^2+^ in this specific case) to Aβ is the indispensable bridge linking the model to the naturally occurring system. Fortunately, the interest in the formation of metal complexes in aqueous solutions has gone beyond the initial purpose of interpreting the structure and the mechanism of formation of a relatively simple complex in solution. Nowadays, studies of the metal-binding affinity of biologically relevant ligands are ubiquitous in bioinorganic chemistry [34,37,51,52,53,54,55,56] and are valuable for the information that they can provide about metal speciation.

Knowing the stability constant values allows to have the species distribution over the pH interval of interest as well as to compare Zn^2+^ affinity for Aβ with that of other ligands. This can provide valuable information on the competition of Aβ for other ligands and vice versa [57]. This is considered of particular interest, as it might be possible to create therapeutic drugs for AD that safely target the Aβ-Zn interaction [43,58]. A thorough speciation might even help explaining why Zn^2+^ inhibits the β-aggregation of both Aβ(1–40) and Aβ(1–42) in a concentration-dependent manner (*vide infra*) [29].

### 3.1. Stability Constant and Speciation

A still debated issue in bioinorganic chemistry is whether metal binding to a given protein site is under thermodynamic or kinetic control [59]. This is a controversial issue as some authors suggest that the metal chemistry of some compartments (e.g., the cytoplasm) is under kinetic control [60], whilst others indicate that metal binding in specific protein sites in vitro is under thermodynamic control [59]. Whatever the situation may be in nature and although the entire process cannot be assumed the summation of the individual steps determining the cascade of events, understanding the thermodynamics of some of the steps involved in the cascade may be crucial to shed light on the entire process. Thus, the thermodynamic characterization of the equilibrium (or the equilibria), eventually reached, becomes the starting point to study a binding process [61]. Unfortunately, the picture for Zn^2+^-amyloid-β affinity is further complicated by the spread of values (and species) reported in literature for Zn^2+^-Aβ binding (Table 1) [34,57,62,63,64,65,66,67].

Although the table contains a few entries, these have all been extracted from only half a dozen papers. Compared to the interaction of Aβ fragments with Cu^2+^ [33] the data available for the analogous interaction with Zn^2+^ are relatively scarce. Perhaps the paucity of data has to do with Zn^2+^ being spectroscopically silent. Please note that the data listed in the table concern different Aβ peptides ranging from Aβ (1–16) to Aβ (1–42). This originates from the commonly accepted view that these peptides retain the binding characteristics of the amyloid-β that cannot be investigated due to its scarce solubility.

All the entries listed in the second column are concentration constants and as such must be retained valid only at or near the conditions at which they were determined [68]. Intentionally, no distinction is made between ^c^K_d_ and ^a^K_d_, i.e., between conditional and apparent constants [26,66,69,70]. It is worth emphasizing, though, that according to the accepted definition the conditional dissociation constant, ^c^K_d_, is the apparent dissociation constant that depends on the pH value and the ionic background employed while ^a^K_d_ is the apparent dissociation constant measured in a given buffer or in the presence of a competing ligand. ^a^K_d_ can easily be converted to ^c^K_d_ by taking into account the competition with other ligand(s), be it a deliberately introduced competing ligand and/or the buffer, if the buffer forms complexes with the metal ion of interest. However, for the sake of clarity, the footnote of Table 1 specifies whether the value refers to a ^c^K_d_ or a ^a^K_d_ according to the source reference. The reader will appreciate that the values significantly depend on the experimental conditions used.

Despite the efforts and the variety of techniques and methodologies employed for the quantification of the metal-protein dissociation constants K_d_, yet there exist significant discrepancies in the literature (Table 1); these may result from the fairly different experimental conditions employed and/or, more likely, from the significantly different concentrations and Zn^2+^/ Aβ ratios explored. The values reported so far range from 1.1 to 300 μM, although there is a general consensus that K_d_ falls in the low micromolar range. Even the stoichiometry of the interactions of Aβ with metal ions is somewhat elusive; in fact, for Zn^2+^-Aβ complexes, stoichiometries ranging from 1:1 to 3:1 have been reported [63,66,71]. Before commenting on the different techniques/methodologies employed to obtain speciation, it is worth delineating a number of issues that must be born in mind when presenting/discussing a K_d_. When using a competing ligand (L) preliminarily evidence should be provided that no ternary (Aβ-M-L or M-Aβ-M’) complex are formed that are the rule rather than the exception. In the context of the present work (Aβ binding to metal ions) this long known concept [72] has been brought up by two research groups recently [73,74].

On the other hand, when working with a high M/L it should also be explored whether binuclear complexes form. An often-overlooked issue is that values of stability constants may be compared only when they refer to species having the same stoichiometry [33,70]. For example, species having the same M/Aβ ratio but a different number of protons (e.g., MAβH and MAβ) cannot be compared; the same applies to species in which the number of protons displaced from the molecule backbone is different (e.g., MAβ and MAβH_-1_) [33]. In connection with the last point, in Table 1 only reference 34 provides absolute values for the binding constants (*vide infra*). We shall briefly discuss the advantages and disadvantages inherent in the determination of absolute and dissociation constants.

To avoid confusion between Aβ and the overall stability constant (conventionally denoted as β) Aβ will be indicated as L throughout the next couple of paragraphs that specifically deal with absolute (stoichiometric) constants; please note that Aβ may denote any fragment of the amyloid-β. We shall assume that *i*. our system contains only one metal cation and one ligand; *ii*. the ligand can take up or release protons; and *iii*. the metal ion and the resulting complexes may hydrolyse. If the metal ion interacts with the ligand in a protic solvent like water, we may write the following equilibrium:

iM + kL + jH ⇆ M_i_L_k_H_j_ (1)

and its associated overall stability constant, $\beta_{M_{i}L_{k}H_{j}}$:

(2) $$\beta_{M_{i}L_{k}H_{j}}=\frac{\left[M_{i}L_{k}H_{j}\right]}{{\left[M\right]}^{i}{\left[L\right]}^{k}{\left[H\right]}^{j}}$$

where [M_i_L_k_H_j_], [M], [H] and [L] are the free concentrations of the complex, the metal ion, the ligand and the proton, respectively (charges are omitted for simplicity). Equation (2) does not represent a thermodynamic stability constant but a stoichiometric stability constant, expressed in terms of concentration quotients, and as such is valid only under the conditions (temperature, pressure, ionic strength) at which is determined whilst thermodynamic constants are dependent only upon temperature and pressure [68]. In order to replace activities, used to express a ‘true’ thermodynamic constant, with concentrations, an inert electrolyte is added. In the presence of relatively large concentrations of “neutral” or inert electrolytes which are assumed not to form complexes with the reacting species, the activity coefficients can be taken as constant. In [M_i_L_k_H_j_] the subscript *j* may have negative values; hence, in the simplest case a species may be represented by the formula MLH_-1_. Such a formula, *per se* ambiguous, indicates that the ML complex has lost a proton, which may either have been released from a water molecule coordinated to the metal ion or from the ligand backbone (e.g., from a peptide nitrogen) if the number of protons that are released exceeds the maximum number of protons that may dissociate from the ligand in the absence of a metal ion. In both cases the species is indicated as [MLH_-1_]; further details on the mathematics behind this may be found in reference [75]. The intentional ambiguity of the MLH_-1_ symbolism is due to the difficulty to identify the origin of the extra-proton that is detected in solution (often by potentiometry, *vide infra*). Note also that if *i* in the expression of β_ikj_, (2), is null, β_ikj_ refers to the overall protonation constant of the ligand. On the other hand, if *k* is null, β_ikj_ will refer to the metal ion hydrolysis; for example, β_10-2_ refers to [M(OH)_2_].

An alternative way to quantify the binding of the metal ion to Aβ is to consider the dissociation equilibrium. In this case, the most common parameter used as a quantitative measure of the binding affinity of a species (e.g., a metal ion) to Aβ is the dissociation constant, K_d_, expressed by the following equilibrium and its related constant:

MAβ ⇆ M’ + Aβ’(3)

(4) $$K_{d}=\frac{\left[\;M\;\prime \right]\left[A\beta \prime \right]}{\left[\mathrm{MA}\beta \right]}$$

where [MAβ], [M’] and [Aβ’] denote the concentration of the MAβ complex, the free concentration of the metal and Aβ, respectively; in equations (3–4) charges are omitted for simplicity. K_d_ is not an absolute constant but is a concentration quotient derived in conditions in which the concentration of one or more reactants is fixed at a particular constant value and thus is strictly valid only for the experimental conditions used, i.e., temperature, pressure, ionic strength, competing ligand (if any) and, much more so, pH. Originally, the concept of ‘apparent/conditional’ stability constant was introduced by Schwartzenbach for EDTA metal complexes [76,77,78] and is used to determine the pH at which EDTA may be employed as a complexing agent for quantitative analysis [79]. It should be noted that [M’] and [Aβ’] are not the parameters defined in Equation (2); they are in fact the total concentrations of free metal ion and Aβ, respectively, present in all their forms at a given pH. It must be emphasized that [Aβ’] is not the concentration of the fully deprotonated ligand but it rather represents an equilibrium mixture of differently protonated ligand species, H_n_Aβ (i.e., Aβ, HAβ, H_2_Aβ ….... H_n_Aβ etc.). Analogously, [M’] denotes the total concentration of the metal ion not bound to Aβ, since the metal ion may hydrolyze and/or interact with a ligand other than Aβ. As detailed above, nowadays in the bioinorganic area a distinction is made between the apparent, ^a^K_d_, and the conditional, ^c^K_d_, constant.

In any case K_d_ proves useful since it allows to consider the complex dissociation as if both M’ and Aβ’ were present under one form only (for a more detailed description please refer to the IUBMB-IUPAC recommendations) [80]. If the system is investigated at a fixed pH, there is no need to determine the protonation constants of the ligand; obviously it holds that there must be no other competing equilibria influencing either [M’] and/or [Aβ’], which must strictly remain constant. If these conditions are not met, K_d_ value will also reflect the changes of [M’] and/or [Aβ’] due, for example, to competing metal hydrolysis and complexation and/or to protonation/deprotonation equilibria. In such cases, corrections should be introduced to take into account the competing equilibria between the metal ion and the ligands (e.g., a competing ligand and/or the buffer).

K_d_ has one undisputable advantage: it gives an idea of the binding affinity of the metal to the biomolecule. In fact, Equation (4) clearly shows that K_d_= [M’] when [Aβ’] is equal to [MAβ]. This means that, when 50% of the initial Aβ is bound to the metal ion, the free metal ion concentration (usually denoted as [M]_50_) is numerically equivalent to the K_d_ value and any procedure leading to the calculation of [M]_50_ may thus provide the K_d_ value [33,54]. It follows that any K_d_ value lesser than the free metal ion concentration of a given physiological compartment implies the formation of significant amounts of MAβ. If the metal ion binds to more than one site within the same biomolecule, [M]_50_ may be considered an ‘average’ of the dissociation constants of each single site. Perhaps it is worth mentioning again that comparisons between K_d_ values determined under seemingly analogous conditions should be avoided as in some cases K_d_ may refer to different species. The potential for error in these studies is high, however, since many competing equilibria may be present even in in vitro solution and must be taken into consideration.

### 3.2. Main Techniques Employed to Determine the Binding Constant

As indicated by Table 1, several techniques have been used to determine the binding affinity of Zn^2+^ to the Aβ. We shall briefly comment on the main techniques (i.e., potentiometry, calorimetry and fluorescence spectroscopy) and highlight the advantages and pitfalls that must be addressed when determining metal–ligand binding constants of biological systems.

#### 3.2.1. Potentiometry

Potentiometry has long been regarded as the most accurate method to determine binding affinities of metal complexes as it provides universally applicable stability constants [81]. With the introduction of accurate and precise glass electrodes, pH-metry has become the technique of choice. It is the only technique that can provide pH-independent stability constants and hence a detailed description of the individual species formed over a relatively large pH interval. It is an indirect technique based on the extra-proton displacement caused by the metal ion. This implies that the protonation constants of the ligand be determined before measuring the actual complexation constant(s). This is not an easy task by itself as a biomolecule may contain several protons that can dissociate in the absence of a metal ion. For instance, ten protonation constants had to be determined for Aβ before proceeding to the investigation of the Zn^2+^-Aβ reported in Table 1 [34,37]. Moreover, it is mandatory that protonation and complexation constants be determined under the same experimental conditions, including ionic strength; usually, the background salt should be one hundred times more concentrated than the reacting species to ensure that coefficients are constant and, thus, justify the use of stoichiometric constants. With the advent of excellent commercially available packages (PSEQUAD [82], HYPERQUAD [75]) data processing and modelling has become increasingly more objective. These packages minimize the function:

U = Σ (X_calc_ − X_obs_)^2^ (5)

where, depending on the program, X may be the analytical concentration, the volume added or the potential; for example, the most commonly used software (HYPERQUAD) minimizes the error square sum in measured potentials. The constants obtained through this procedure express the explicit metal and proton stoichiometries of complexes. Unfortunately, this technique requires millimolar solutions. Though accurate, this methodology is time consuming since several titrations must be carried out to determine both protonation and complexation constants; in addition, compared with other techniques, it requires relatively large concentrations of biomolecule, which may pose solubility and cost problems.

#### 3.2.2. Calorimetry

In the last decade, isothermal titration calorimetry (ITC) has been used to determine K_d_ [26,57,70,83]. ITC is particularly suitable for the study of the interactions of biomolecules with spectroscopically silent metal ions like Zn^2+^ that lack traditional spectroscopic signatures characteristic of other metal ions associated with d–d transitions [84]. The introduction of calorimeters, that make use of small volume cells and have fairly low detection limits by Microcal (now Malvern) and Calorimetry Science Corporation (now TA Instruments), determined the surge in popularity for the study of chemical binding phenomena and the widespread use of isothermal titration calorimetry [61,85,86,87]. Calorimeters belonging to this class directly produce the time derivative of the thermogram (dQ/dt vs. time) that can be integrated over time to give the heat produced or absorbed during the chosen time interval. Since ITC experiments provide a quantity, the gross heat (Q), that includes a number of additive terms, they need to be carefully designed to obtain the net heat of reaction. Suggestions/advices to avoid the pitfalls concerning the production of good quality data can be found in references [61,84,85,86,87].

The determinability and the accuracy of K, ΔH, and the stoichiometry factor n basically depend on the so-called Wiseman ‘c’ value:

c = n K_f_C_R_ (6)

where n, K_f_ and C_R_ are the stoichiometric factor, the binding constant and the total macromolecule concentration [88]. The ‘c’ value should fall in the range 10 < c < 1000. A more recent treatment linking properties of the reaction (*K*_f_ and ∆H) and properties of the calorimeter (V_R_ and δQ) demonstrates that the following condition:

*K*_f_/│∆H│ < 4.72 V_R_/δQ (7)

should be satisfied, where V_R_ and δQ are the active reaction volume of the cell and the uncertainty in the heat per data point in the titration, respectively [86]; this narrows down the Wiseman window and thus the c value should range from 50 to 500. However, the lower boundary is still a matter of debate [61,89,90]. Like in potentiometry, the function U is minimized:

U = Σ (Q_exp,corr_ − Q_calc_)^2^ (8)

where Q_exp,corr_ is the experimental value of the ‘net’ heat generated in a reaction step (*vide infra*). Q_calc_, is related to the change, δn_i_, in the number of moles of the *i*-th chemical species by Equation (9):

(9) $$Q_{calc}=-\overset{n}{\underset{i=1}{\sum }}\delta n_{i}{\Delta H}_{i}$$

δn_i_ values, that represent the change in the number of moles of the *i*-th reaction component, are calculated with a given set of stability constants and experimental conditions.

As ITC experiments are often conducted at constant pH they do not require prior knowledge of protonation constants. By contrast, only ‘apparent’ constants are produced; best fit yielding non-integer stoichiometries (e.g., 1.5), whose interpretation is rather puzzling, are sometimes reported [57]. Apparent constants can be turned into ‘conditional’ constants but this introduces a further degree of uncertainty due to error propagation. In any case, the ‘apparent’ constant thus obtained does not refer to a specific species.

Recently, a new package of the HYPERQUAD suite, HypCal, has been published that can provide stoichiometric (absolute) formation rather than ‘apparent’ dissociation constants [91]. Although calorimetry does not require the prior knowledge of a protein pK_a_ values, can provide K, ΔH and ΔS values and is in principle applicable to larger molecules than potentiometry it suffers from major drawbacks. The physical quantity measured (heat, Q_exp_) must be corrected for all non-chemical energy terms (stirring, dilution of titrant and titrate, etc.) to obtain the ‘net heat’ value, Q_exp,corr_, before processing the data. Secondly, the heat contribution resulting from the interaction with components of the solution other than the analyte (e.g., a competing ligand employed in a titration, the buffer, hydrolysis of the metal ion) must be precisely known in the conditions employed for the actual titration under study (i.e., temperature, ionic background).

#### 3.2.3. Spectroscopy

Spectroscopic titrations may also be used to quantitate Zn^2+^ binding to Aβ. Despite the similarity between spectroscopic and calorimetric titrations was evidenced by Bolles et al. long ago [92], only recently has UV-Vis spectroscopy been used to determine the apparent zinc association constant to Aβ [67]. Unlike calorimetry, spectroscopy follows the change of signals directly and necessitates concentrations lower than those used in calorimetric experiments. Although spectroscopy has been used to determine stoichiometric association constants in relatively complex systems [93] has not gained much popularity in metal–biomolecule studies. This is surprising if one considers that packages that can provide stoichiometric constants like PSEQUAD [82], HYPERQUAD [75], SPECFIT [94] have been available for some time now; incidentally, these programs utilize a multi-wavelength treatment of spectral data thus minimizing the risks of creating artifacts associated with a single-wavelength treatment. Aβ has a fluorophore (tyrosine in position 10) that can be exploited to run spectrofluorometric titrations. In spectrofluorometric titrations signals may be followed directly too, with the additional advantage that very low concentrations can be explored. Titrations based on the intrinsic tyrosine fluorescence [64,66] as well as competition experiments [57,65] have been used to determine Zn^2+^ binding to Aβ. However, both types of experiments were used to determine apparent and not stoichiometric constants.

Job plots often obtained from spectroscopic experiments are only indicative as they can conceal contributions resulting from additional species that are formed in the same mixture. Garzon-Rodriguez et al., who used this method to model Zn^2+^ and Cu^2+^ complexation with Aβ(1–40), Aβ(1–42) and an Aβ(1–40) fragment having a tryptophan in position four, clearly state that the Job plots obtained via fluorescence measurements show maxima corresponding to peptide-to-metal stoichiometry between 1:1 and 1:2. Incidentally, the Job plots for Cu^2+^ ion showed the stoichiometry closer to 1:1 with some possible contribution from a 2:1 peptide-metal complex, which again highlights the limits of this methodology [64].

#### 3.2.4. NMR

NMR may also be used to determine the association constant(s). However, this technique requires relatively large amounts of material; furthermore, the buffer (if any) must be deuterated in ^1^H-NMR studies, and this may render the method fairly expensive. Moreover, people who have been extensively using this technique explicitly state that K_d_ resulting from their NMR study is not a quantitative measure of the binding of zinc by the molecule but rather a measure of which residues are most involved in the binding [65].

#### 3.2.5. Other Techniques

In addition to those briefly illustrated above, in principle other techniques (e.g., CD, EPR and ESI-MS) might be used to determine the association constant(s). Like UV-vis and fluorescence, CD requires that the bands of each individual complex differ from each other enough to allow for the determination of the concentration of each complex species. As this is not often the case, CD as well as EPR have been used to validate the model obtained through potentiometric measurements.

ESI-MS involves a ‘significant alteration of the physical environment of the reaction studied’ (i.e., transition to the gas phase) and thus requires validation for the specific reaction studied or combination with other techniques [70,95]. For example, within the framework of Zn-Aβ interaction/speciation, Damante et al. have combined potentiometric, NMR, and ESI-MS investigations to demonstrate that the Aβ(1–16) is able to coordinate up to three zinc ions [34].

### 3.3. Use of Speciation Data

The data listed in Table 1 shows that to date there have been many inconsistencies in the literature on both the affinity of Zn^2+^ for Aβ as well as on the stoichiometry of the species resulting from Zn^2+^ interaction with Aβ peptides. Bush et al. reported on a highly specific pH-dependent high- and a low-affinity binding; they also reported different Zn^2+^: Aβ ratios (stoichiometry) for such low and high affinity bindings [62] derived from Scatchard plots. The existence of a high- and low-affinity binding site is also reported by Danielsson et al. [65], based on NMR results, whilst Clements et al. found no evidence for the higher affinity binding site [63]. Based on Job-plots obtained via fluorescence measurements Garzon-Rodriguez et al. reported for Zn^2+^ a peptide-to-metal stoichiometry between 1:1 and 1:2 while they found no evidence for a high affinity binding [64]. Tougu et al. highlighted that the ‘interaction of Zn^2+^ with the amyloid peptides cannot be characterized by a single conditional K_d_ value’ and stressed that likely ‘in the case of zinc the K_d_ value does not belong to a single and well-defined Zn^2+^-Aβ complex’ [66]. Clements et al. pointed out that the composition of the buffer, the different experimental conditions employed and/or, more likely the significantly different Zn/Aβ ratio explored may be responsible for some of the above discrepancies [63]. It has been reported that high concentrations of metals ions, like zinc, copper and iron, may induce A*β* aggregation [22,23] and fibril formation [24,25], while low concentrations of zinc and copper selectively lower the highly toxic A*β* oligomeric species [20,50]. Zawisza et al. underlined that the range of values of stability constants which can be determined by the different methods may be the source of significant controversies, particularly in Aβ research. They also pointed out that ‘the description of the coordination process in terms of sets of pH-independent cumulative stability constants’, as those expressed by Equation (2), is the only method that can describe individual components of the chemical equilibrium under given conditions [70].

Though different authors [34,57,62,63,64,65,66,67,70] have attempted to explain/reconcile the diverging data concerning Zn^2+^ affinity constants for Aβ, the correlation between zinc-binding affinity, metal coordination features, the morphology of zinc-containing aggregates and their different toxicity are all still matter of discussion.

In the examples that follow, we show that the determination of the stoichiometry of the Zn^2+^ complexes (mono-, bi-nuclear, protonated, hydroxo-complexes) as well as of their stability constants over a wide pH range, may allow to correlate a given property with a specific species that is formed in determined conditions (pH, buffer, competing ligand, etc.); this may avoid, for example, to correlate results obtained through different techniques (and experimental conditions) with ‘high-’ and/or ‘low-affinity’, which very likely reflect a mixture of Zn^2+^-Aβ species. Perhaps it is worthy stressing that the methods and the procedures used for evaluation of binding constant values for the Zn^2+^-Aβ peptides actually measure average parameters of the mixture of complexes with the exception of potentiometric studies. Moreover, the most common techniques briefly reviewed above make use of sizably different concentrations which implies the formation of different chemical species and may account, at least in part, for the spread of the values listed in Table 1.

**Figure 2:**
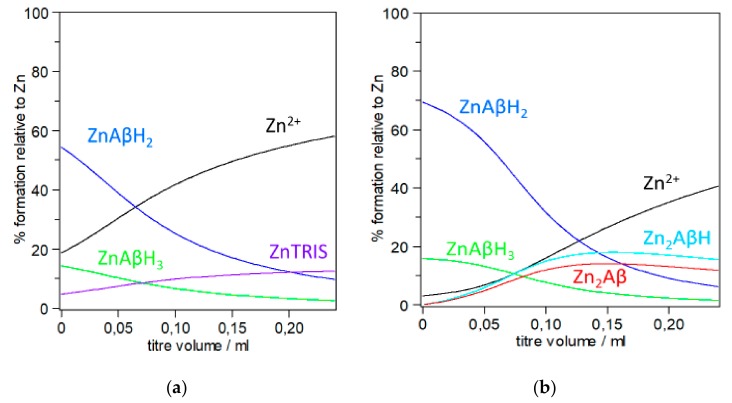
Computed species distribution reproducing the addition of Zn^2+^ to Aβ(1–16)PEG in 20 mM TRIS buffer, 100 mM NaCl, pH 7.4, t = 25 °C; (**a**) C_Zn_ = 5 × 10^−4^, C_Aβ_ = 20 μM; Zn^2+^ (black line), ZnAβH_3_ (green line), ZnAβH_2_ (blue line), ZnTRIS (fuchsia line); (**b**) C_Zn_ = 3 × 10^−3^ M, C_Aβ_ = 140 μM; free Zn^2+^ (black line), ZnAβH_3_ (green line), ZnAβH_2_ (blue line), Zn_2_AβH (turquoise line), Zn_2_Aβ (red line). Aβ(1–16)PEG protonation constants from [37]; TRIS protonation and Zinc-TRIS formation constants and Zn^2+^ hydrolysis constants from [96]. Species percentage was computed by using HYSS [97].

In the first example that reproduces two ITC titrations run with different Zn^2+^ and Aβ concentrations we highlight the problems associated with the use of apparent binding constant instead of stoichiometric constants (Figure 2). For the sake of simplicity, reactant concentrations (and conditions) were taken from reference [57], although the discussion that follows might be extended to similar experiments, whose conclusions are based on K_d_. For the sake of visual clarity, species forming below ten percent are not plotted in the figures. The computation of the species distribution has been carried out by using the only set of stoichiometric constants available in the literature [34].

Figure 2 prompts a few considerations that are crucial to the correlation of results with the species actually existing in solution. Figure 2a,b show that multiple species are formed in both cases. This strongly supports the assertion by Togu et al. that in the case of ‘zinc the ^a^K_d_ value likely does not belong to a single and well-defined Zn^2+^-Aβ complex’ [66]; this view is shared by Damante et al. who, based on a multi-technique investigation, have also highlighted that the data obtained just below pH 7 cannot be attributed to a single complex species [34]. A second consideration focuses on the significant difference between the two titrations depicted in Figure 2a,b. Only mononuclear zinc complexes ([Zn(Aβ)H_3_] and [Zn(Aβ)H_2_]) are formed in Aβ more dilute solutions (Figure 2a), while binuclear Zn^2+^ species also form in more concentrated Aβ solutions (Figure 2b). Furthermore, although both the mononuclear species of Figure 2a have the same Zn/Aβ ratio (1:1) their contribution to the total measured heat is unlikely to be the same since they have a different number of protons. Moreover, a complex with the buffer tends to form in the final region of the titration (~12%), which will contribute to the total heat (Figure 2a). The nature of the buffer, its concentration as well as the peptide/buffer ratio opens a new chapter since the buffer may act as a competitive metal-binding agent and as such often has an appreciable effect on the metal speciation in solution. In the effective words of Magyar et al., buffers are ‘non-innocent’ components of the solution investigated in vitro [59]. Togu et al. have highlighted that ^a^K_d_ for Zn^2+^-Aβ complex is higher at higher concentrations of TRIS, while there is no difference between the ^a^K_d_ values in 20 mmol/L HEPES, and in 10 mmol/L TRIS [66]. Johnson et al. have pointed out that buffers can significantly affect the interpretation of thermodynamic data; they have also provided a good example illustrating how complexes with and proton transfer to a buffer may be accounted for [84]. Taking into account the contribution of the complex formed with the buffer to the measured physical quantity (Q_exp_) might help at least in part unravelling some of the discrepancies reported in the literature. The presence of a buffer, while particularly relevant for some techniques (e.g., ITC), may also affect the interpretation of results obtained through spectroscopic experiments (e.g., NMR). For example, commenting on their NMR data, Danielson et al. have underlined that ‘quantitative results may be somewhat biased, due to metal–phosphate complex‘, i.e., the buffer used in their experiments [65]. In more concentrated solutions (Figure 2b), binuclear species ([Zn_2_(Aβ)] and [Zn_2_(Aβ)H]) are also formed which together total ca. 30% of total zinc at the end of the titration while in the same region the zinc complex with the buffer is just below 10% (~8%, not shown in the figure). The formation of multiple coexisting species probably accounts for the non-integer stoichiometries obtained when fitting the ITC data obtained for the titration represented in Figure 2b as well as for the different number of protons transferred to the buffer when zinc is added to a peptide solution or when the peptide is added to a zinc solution [57]. Noteworthily, even in regions where the total Zn^2+^ over the total Aβ concentration is approximately equal to 1.5 (i.e., titre = 0.12), the percentage of Zn^2+^ binuclear species that are formed roughly matches that of the mononuclear species. Thus, assumptions on the Zn/Aβ ratio, often referred to as stoichiometry, of the species resulting from the binding of the metal ion to Aβ fragments should be made with extreme caution as the interaction is more complex than it might seem at first sight. Perhaps, the relevance of binuclear species is even more evident if we consider that the synapse is the main arena of molecular events that lead to neurodegenerative disorders involving Aβ peptides and that Zn^2+^, as well as other metal ions, is released into the synaptic cleft in the process of Glu-mediated neurotransmission. Zawisza et al. have figured out that the metal ion concentrations in the glutamatergic cleft is of the order of 0.1 mM and, locally and temporarily, might be even higher at the site of release [70]. It has to be evidenced that at the end of the run represented in Figure 2a,b, the Zn/Aβ ratio is about 4.5 and 3, respectively. The amount of ‘free’ Zn^2+^ calculated for the in vitro experiments depicted in Figure 2 should not lead to the conclusion that free Zn^2+^ exists in vivo since in human zinc metabolism, several ligands (e.g., low molecular weight ligands, proteins) participate in cellular uptake, extrusion, and re-distribution [98]. The potential for misinterpretation in metal ion-peptide studies is high since many competing equilibria are present in solution and this further stresses the necessity to have stoichiometric constants as they can provide valuable information about metal speciation and exchange in biological systems [33,59].

Incidentally, a ubiquitous ligand, OH^-^, is often overlooked. The hydroxo ion may be of paramount importance since all measurements in vitro tend to reproduce naturally occurring conditions and are thus carried out at or around physiological pH values; at these pH values, even if in the presence of a strongly coordinating ligand OH^-^ does not cause the formation of metal ion hydroxide, more often than not comes in as a ‘third’ ligand thereby leading to the formation of hydroxo species of the type M_i_L_k_(OH)_j_. In cases where stoichiometric constants cannot be obtained, due the complexity of biological systems, we as well as other authors emphasize that the characteristics of complexes should never be compared unless it can be proved that they have the same stoichiometries [33,70].

In the second example, we deal with an interesting result, reported by Yoshiike et al., who in an effort to see whether some metal ions (*viz.* Zn^2+^ and Cu^2+^) promote or inhibit the formation of aggregates, performed a series of experiments on Aβ(1–40) and Aβ(1–42) by using different techniques and methods [29]. Based on fluorescence, UV, CD, cell culture experiments, they reported that ‘Zn(II) and, to a lesser extent, Cu(II) prevent Aβ from forming β-sheet conformations in an as-yet-undetermined manner’, evidencing that ‘with concentrations greater than 10 μM, both Zn(II) and Cu(II) effectively suppressed β-aggregation, resulting in an increase in cell viability’. Indeed, conflicting views on whether Zn^2+^ has beneficial or detrimental effects have been reported [22,26,27,28,29,30,31,32]. A speciation based on the use of absolute (stoichiometric) stability constants might help explaining why in the experiments reported in [29], Zn^2+^ inhibits the β-aggregation of both Aβ(1–40) and Aβ(1–42) in a concentration-dependent manner. To this end, we computed the species distribution with a view to modelling the intriguing effect of Zn^2+^ on the inhibition of aggregates reported by Yoshiike et al. [29]. These authors explored *inter alia* Zn^2+^ concentrations ranging from zero up to 100 μM in a medium containing 5 μM Aβ(1–40) in order to understand why low Zn^2+^ concentrations decrease the formation of toxic fibrils whilst concentrations greater than 10 μM inhibit the formation of β-aggregates and increase cell viability; interestingly it was also reported that the effect of Zn^2+^ concentration tends to level off above 40 μM.

**Figure 3:**
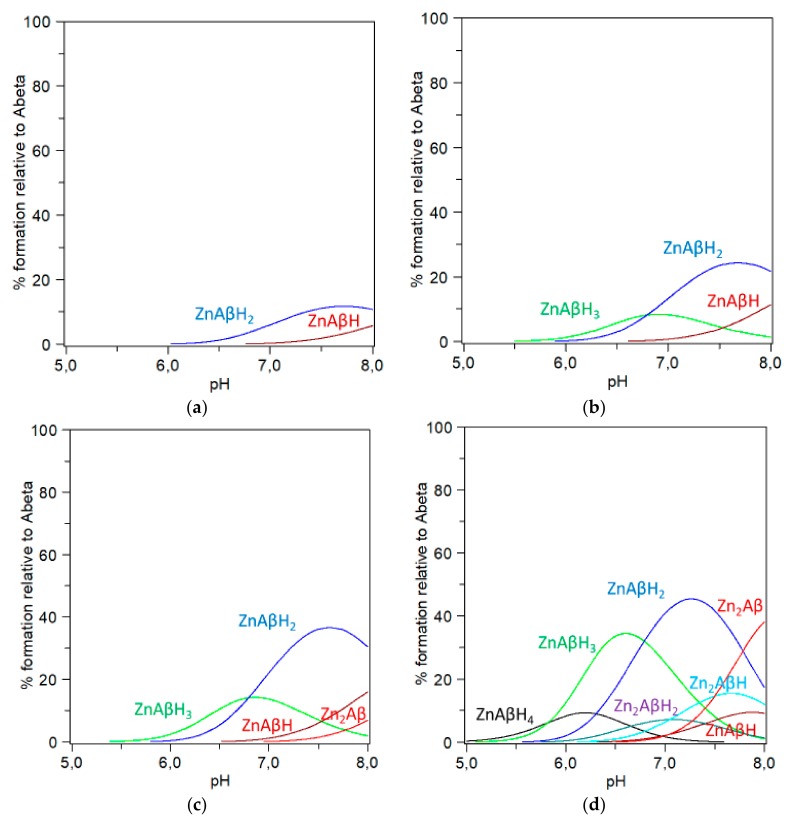
Computed species distribution mimicking the assays by Yoshiike et al. [29] who investigated the concentration dependent inhibition of β-aggregates (ThT test) and the increase of cell (MTT test). Figure 3a–d reproduce the addition of Zn^2+^ to Aβ(1–16)PEG in 20 mM TRIS buffer at pH 7.4, t = 25 °C; C_Aβ_ = 5 μM in all diagrams; (**a**) C_Zn_ = 2 μM; ZnAβH_2_ (blue line), ZnAβH (amaranth line); (**b**) C_Zn_ = 5 μM, ZnAβH_3_ (green line), ZnAβH_2_ (blue line), ZnAβH (amaranth line); (**c**) C_Zn_ = 10 μM, ZnAβH_3_ (green line), ZnAβH_2_ (blue line), ZnAβH (amaranth line), Zn_2_Aβ (red line); (**d**) C_Zn_ = 70 μM, ZnAβH_4_ (black line), ZnAβH_3_ (green line), ZnAβH_2_ (blue line), ZnAβH (amaranth line), Zn_2_AβH_2_ (fuchsia line), Zn_2_AβH (turquoise line), Zn_2_Aβ (red line). Aβ(1–16) PEG protonation constants from reference [37]; TRIS protonation, Zinc-TRIS formation and Zn^2+^ hydrolysis constant from reference [96]. Species percentage was computed by using HYSS [97].

Similarly to what we did to obtain the species distribution for the examples shown in Figure 2, we used the set of data reported in literature for the representative Aβ(1–16) PEG peptide and investigated Zn^2+^ concentrations ranging from 0.05 to 70 μM (0,05, 2, 5, 10, 20, 30, 40, 50, 60 and 70 μM) while keeping Aβ concentration constant (5 μM) as done by Yoshiike et al. in their experiment on Aβ(1–40) [29]. Figure 3a–c and d show selected species distribution obtained at 2, 5, 10 and 70 μM, respectively.

Species whose formation is less than five percent are not shown. Figure 3a–d show neither ‘free’ Zn^2+^ (not bound to Aβ) nor ZnTRIS; the latter species is just above thirty percent (relative to total zinc) in the conditions depicted in Figure 3d. Figure 3a,b show that sizable amounts of Zn^2+^-Aβ complex species are formed as the Zn/Aβ ratio reaches the 1:1 value (i.e., 5 μM, Figure 3b). As pointed out for the experiments reproduced in Figure 2a,b, the picture is more complex that one may think. The species distribution strengthens the view that in the case of zinc the K_d_ value does not belong to a single and well-defined Zn^2+^-Aβ complex [66] but is rather represented by a complex mixture that above a certain Zn^2+^/Aβ ratio involves both mono- and bi-nuclear species [34]. Noteworthy, significant percentages of zinc species are formed ‘with concentrations greater than 10 μM’ and Zn/Aβ ratios equal to or greater than two, i.e., at the concentration where zinc effectively suppressed β-aggregation (ThT results in [29]), and increased cell viability (MTT results in [29]).

Moreover, the species distribution shown in Figure 3 helps explain why zinc-promoted non-β-sheet aggregates are destabilized when pH lowers slightly as found around the site of inflammation [29]. In fact, at pH = 6.8 the percentage of Zn^2+^-Aβ species is sizably smaller than that detected at pH 7.4 and this supports the hypothesis that an ‘aberrant increase in β-aggregation in the presence of zinc may represent the pathological mechanism by which acidosis induces the liberation of Aβ from Zn(II)’ [29].

**Figure 4:**
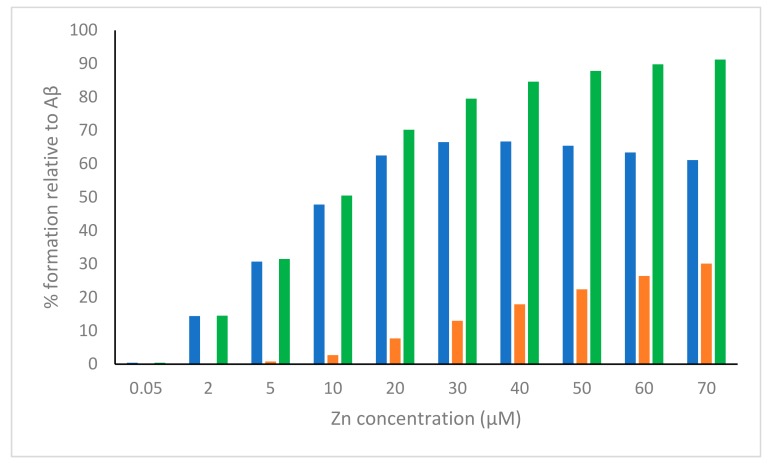
Total percentage of mono-, bi-nuclear species computed at pH = 7.4 for the experiments reported in Figure 6 of [29]. Blue, red and green represent the total percentage of all mono-nuclear, bi-nuclear and mono- plus bi-nuclear species, respectively.

In order to have an overview of both the mono- and bi-nuclear species formed over the range explored and to find a possible link between the species and the effect observed, we have reported the sum of the percentages of mono-, bi-nuclear zinc species and Zn-TRIS versus the concentration of Zn^2+^ added (Figure 4).

For the sake of visual clarity, Figure 4 does not show the percentage of the species resulting from the interaction of Zn^2+^ with the buffer (relative to zinc concentration) calculated for each Zn^2+^ concentration (0.05, 2, 5 μM etc.). As anticipated, such a percentage is just above 20% at 0.05 μM increases to about 30% at 70 μM, and may be retained constant over the entire interval, considering the wide range of zinc concentrations and Zn/Aβ ratios investigated. Figure 4 would indicate that the effective suppression of β-aggregation and the consequent increase in cell viability does not depend on zinc concentration as much as on the amount of Zn-Aβ species formed. The trend represented by the sum of zinc mono-and binuclear species (green bars) levels off and mirrors that observed by Yoshiike et al. in their experiments. Although a more confident statement about the physiological significance of the link between speciation and effect would require a more detailed study, it might be speculated that the main species that are formed in in the presence of excess Zn^2+^ might be responsible for both the inhibition of the formation of β-aggregates as well as the increase of cellular viability. Overall, our data support the idea that Zn^2+^ and much more so Zn^2+^-Aβ species have a protective role against β-amyloid toxicity [26,29].

## 4. Concluding Remarks

It must be underlined that there is nothing wrong with the determination of an apparent (K_d_) constant and often this is the only stability constant that may be accessed experimentally at a given pH value when for instance, neither the protonation constants nor the speciation of the metal ion with the protein (or fragments of the protein) are available. However, we emphasize that using an apparent Kd to draw conclusions on the structure of the complex species formed may be misleading since, as underlined when commenting the terms (M’ and Aβ’) that appear in the equation expressing K_d_ (Eq. 4), M’ and Aβ’are in fact the total concentrations of free metal ion and free Aβ, respectively, present in all their forms at a given pH. As a consequence, the apparent ^a^K_d_ should be used only to compare values measured under the same conditions and in particular the same buffer and the same concentration of the buffer [26,66].

The determination of stoichiometric constants (where feasible) can be a time-consuming and tedious procedure, since numerous titrations under various conditions (different concentrations as well as different metal/Aβ ratios) must be carried out to avoid bias and to ensure that the calculated stoichiometric constants provide, in fact, the best descriptions of the system. However, these efforts may be rewarding; as emphasized by Magyar et al. as the binding interactions of the peptide(s) with metal ions are described on a truly quantitative basis we will gain a much greater understanding of the roles and relationships of metals in biology and biological processes [59].

If the experiment to measure the binding constant is well planned and all the parameters (pH, ionic strength, buffer etc.) influencing this thermodynamic quantity are duly taken into account, Einstein’s statement that reads

‘A theory is the more impressive the greater the simplicity of its premise is, the more different kinds of things it relates, and the more extended is its area of applicability. It [thermodynamics] is the only physical theory of universal content concerning which I am convinced that, within the framework of applicability of its basic concepts it will never be overthrown’ will be more appropriate than ever [99].

## Figures and Tables

**Table 1:** Literature values for Zn^2+^ binding to Aβ fragments.

Aβ Fragment ^a^	K_d_ (μM)	pH	T (°C)	Conc. (μM)	Method	Buffer	Background Salt	Ref.
1–40	5/0.1	7.4	20	-	Radioact. Sat.bind.	TRIS (20mM)	0.1 M NaCl + 1mM MnCl_2_	62
1–40	3.5	7.4	20	-	Radioact. Sat.bind.	TRIS (50mM)	1 M KCl	63
1–40	300	7.4	n.s.^b^	3	Tyr. fl.	TRIS/HEPES (10 mM)	0.1 M NaCl	64
1–42	57	7.4	n.s.^b^	3	Tyr. fl.	TRIS (10 mM)	0.1 M NaCl	64
1–28	1.1	7.2	20	10	Tyr. fl. Zn/Cu Compet.	Phosphate (10 mM)	none	65
1–40	1.2	7.2	20	50	NMR	Phosphate (10 mM)	none	65
1–28	6.6	7.2	20	10	Tyr. fl. Zn/Cu Compet.	HEPES (10 mM)	none	65
1–16	22/71 ^c,d^	7.4	25	20/140 ^c^	ITC	HEPES/TRIS (20 mM) ^e^	0.1 M NaCl	57
1–28	10/30 ^c,d^	7.4	25	20/140 ^c^	ITC	HEPES/TRIS (20 mM) ^e^	0.1 M NaCl	57
1–40	7/3 ^c^	7.4	25	10/70 ^c^	ITC	HEPES/TRIS ^e^ (20 mM) ^e^	0.1 M NaCl	57
1–16	14	7.4	n.s.^b^	10	Fl. Zincon Compet.	HEPES (20 mM)	0.1 M NaCl	57
1–28	12	7.4	n.s.^b^	10	Fl. Zincon Compet.	HEPES (20 mM)	0.1 M NaCl	57
1–40/1–42	7/7	7.4	n.s. ^b^	10	Fl. Zincon Compet.	HEPES (20 mM)	0.1 M NaCl	57
1–40	65	7.4	n.s. ^b^	4	Tyr. fl.	HEPES (20 mM)	0.1 M NaCl	66
1–42	91	7.4	n.s. ^b^	4	Tyr. fl.	HEPES (20 mM)	0.1 M NaCl	66
1–40	60	7.4	n.s. ^b^	4	Tyr. fl.	TRIS (10 mM)	0.1 M NaCl	66
1–40	184	7.4	n.s. ^b^	4	Tyr. fl.	TRIS (100 mM)	0.1 M NaCl	66
1–40	11/2 ^f^	7.3	n.s. ^b^	12	Fl. Zincon Compet.	HEPES (50 mM)	0.1 M NaCl	66
1–16-PEG	- ^g^	-	25	1–4 (×10^3^)	Potentiometry	No Buffer	0.2 M KCl	34
1–16	9	7.1	25	- ^h^	UV-Vis Compet.^h^	HEPES (50 mM)	none	67

a. Only data for soluble fragments are shown in the table; b. not specified; c. the two values were obtained by using the ‘low’ and ‘high’ concentrations shown in the adjacent column; d. the best fit yielded a stoichiometry of about 1.5; e. experiments were also run by using cacodylate buffer; f. the two values were obtained by competition with Zincon after incubation for 3 and 30 min, respectively; g. no value is reported as the best model contains more than one Zn^2+^ complex near neutrality- ten protonation constants are reported; h. determined by UV-Vis competition experiments with a new water-soluble Zn^2+^ chelator-Aβ was added to a solution of the chelator (60 μM) and Zn^2+^ (50 μM), final Aβ/Zn^2+^ ratio was 10/1.
